# Treatment Outcome and Mortality at One and Half Year Follow-Up of HIV Infected TB Patients Under TB Control Programme in a District of South India

**DOI:** 10.1371/journal.pone.0021008

**Published:** 2011-07-26

**Authors:** Sophia Vijay, Prahlad Kumar, Lakbir Singh Chauhan, Saroja Vadigepalli Narayan Rao, Preetish Vaidyanathan

**Affiliations:** 1 National Tuberculosis Institute, Bangalore, India; 2 Central Tuberculosis Division, Directorate General of Health Services, Ministry of Health and Family Welfare, New Delhi, India; McGill University, Canada

## Abstract

**Background:**

There is paucity of data from India on the impact of HIV related immunosuppression in response to TB treatment and mortality among HIV infected TB patients. We assessed the TB treatment outcome and mortality in a cohort of HIV infected TB patients treated with intermittent short course chemotherapy under TB control programme in a high HIV prevalent district of south India.

**Methodology/ Findings:**

Among 3798 TB patients registered for treatment in Mysore district from July 2007 to June 2008, 281 HIV infected patients formed the study group. The socio-demographic and treatment related data of these patients was obtained from TB and HIV programme records and patient interviews 19 months after TB treatment initiation by field investigators. Treatment success rate of 281 patients was 75% while in smear positive pulmonary tuberculosis cases it was 62%, attributable to defaults (16%) and deaths (19%). Only 2 patients had treatment failure. Overall, 83 (30%) patients were reported dead; 26 while on treatment and 57 after TB treatment. Association of treatment related factors with treatment outcome and survival status was studied through logistic regression analysis. Factors significantly associated with ‘unfavourable outcome’ were disease classification as **Pulmonary** [aOR-1.96, CI (1.02–3.77)], type of patient as **retreatment** [aOR-4.78, CI (2.12–10.76)], and **non initiation of ART** [aOR-4.90, CI (1.85–12.96)]. Factors associated with ‘Death’ were non initiation of ART [aOR-2.80, CI (1.15–6.81)] and CPT [aOR-3.46, CI (1.47–8.14)].

**Conclusion:**

Despite the treatment success of 75% the high mortality (30%) in the study group is a matter of concern and needs immediate intervention. Non initiation of ART has emerged as a high risk factor for unfavourable treatment outcome and mortality. These findings underscore the importance of expanding and improving delivery of ART services as a priority and reconsideration of the programme guidelines for ART initiation in HIV infected TB patients.

## Introduction

TB is the commonest Opportunistic Infection (OI) among HIV infected patients, particularly in the developing countries [1], [2]. HIV fuels progression to active disease in people infected with TB with an annual risk of 5–15% compared with 10–20% life time risk among non HIV infected patients [3]. The HIV/AIDS epidemic has a dramatic impact on the epidemiology of tuberculosis. This has increased the global tuberculosis burden [4] especially in populations where HIV is common and where the prevalence of tuberculosis infection is high [5], [6]. The overlap of HIV and TB epidemics is particularly striking in south East Asian countries. HIV prevalence among TB patients is higher than in general population which has been documented in several studies from different parts of the country [7].

India has the world's highest burden of tuberculosis, with 2 million (1.6–2.4 million) incident cases, an estimated 6.4% (3.9–9.8%) are HIV infected and has 2.3 million persons living with HIV/AIDS [8], [9]. The policy of co-ordinated TB and HIV-AIDS control programme activities makes early detection of HIV infection among TB patients possible and also offers an opportunity to promptly link patients to HIV care interventions. We conducted a study in 2007 in two districts of south India to evaluate the feasibility of provider initiated HIV testing and counselling (PITC) of TB patients and to assess the efficiency of linkage of HIV sero-positives for care and treatment [10]. This study provided the first evidence from India on the successful implementation of PITC services for TB patients by the general health care system in high prevalence settings. However, the linkage to life saving Anti Retroviral Treatment (ART) services was found lacking.

Tuberculosis is still the leading cause of mortality among HIV infected patients and this accounts for one third of the deaths due to AIDS worldwide [11], [12]. Striking impact of HIV infection on mortality among TB patients has been reported from several studies [13]–[17]. Though, mortality rate from HIV associated TB in developing countries is high, it is not clear whether it is due to failure of anti TB treatment or complications of HIV [15].

There is paucity of data from India on the impact of HIV related immunosuppression on response to TB treatment and subsequent mortality [18]. This information is essential for implementing interventions to improve the survival of HIV infected TB patients. We followed up the cohort of HIV infected TB patients of our earlier study after their treatment with Intermittent Short Course Chemotherapy (SCC) regimen under the TB control programme. The primary objective was to assess their TB treatment outcomes and mortality after treatment. The secondary objective was to identify treatment related factors associated with TB treatment outcome and survival status.

## Methods

### Ethics statement

The purpose of seeking information was explicitly told to the patient. Individual informed verbal consent was obtained prior to interview as there was no intervention / procedure involved. Patient's consent to participate was recorded on the study format. Data collected from patients and records were confidentially maintained by the study staff. The study procedures including verbal consent from patient were approved by the ethics committee of the National Tuberculosis Institute, Bangalore. Approval and financial support for the research was granted by the Ministry of Health and Family Welfare, Government of India.

### Setting

The study was conducted in Mysore district of Karnataka state (population 2.8 million). The district has been classified as high priority for HIV interventions by National AIDS Control Organization (NACO) on the basis of consistently having >1% HIV seroprevalence during sentinel surveillance at antenatal clinics [19]. In the district, tuberculosis control programme services are available through a decentralized network of primary health care facilities which provide general health services including quality-assured smear microscopy and directly observed treatment through health facilities and community DOT providers. All TB patients initiated on treatment were registered at the six sub district level TB programme management units. Voluntary HIV counselling and testing services were offered through National AIDS Control Programme (NACP) - supported network of 27 integrated counselling and testing centres (ICTC). Free antiretroviral treatment (ART) was provided at one ART centre, where HIV-infected patients are screened for ART eligibility and offered HIV treatment and care. These service delivery sites under NACP follow the national guidelines for counselling, testing, care and treatment of HIV-infected patients [20].

### Design and Definitions

In this observational study a cohort of TB patients registered for treatment in the district was followed up prospectively. The end points assessed were TB treatment outcomes and survival status after treatment period of HIV infected TB patients in the cohort. The TB treatment outcomes were assessed as ‘favourable outcome’ (cure and treatment completed) and ‘unfavourable outcome’ (default, dead, failure). Retreatment cases were those having history of previous TB treatment of >1month. Treatment regularity is defined as patient not missing any dose either in the intensive or continuation phase of treatment.

### Study group

In the cohort of 3798 TB patients registered under the Revised National Tuberculosis control Programme (RNTCP) from 1^st^ July 2007 to 30^th^ June 2008, in the district, 72% were aware of their HIV status and of them 281 patients identified as HIV infected during our earlier study constituted the **study group**. These patients were treated with intermittent SCC regimen administered under DOT following the programme guidelines [21]. After an average period of one and half year of TB treatment initiation, the study group was contacted and interviewed to collect the required information.

### Data collection and analysis

The data sources for the study were RNTCP and NACP records and details from the patient interviews. Patients were interviewed in local language by trained field investigators using a pre-tested questionnaire. To ensure adequate coverage minimum three attempts were made to approach the patients.

The line list of the study cohort was available from the earlier study. This line list elaborating details regarding TB treatment, referral to ICTC and linkage to ART was also referred to abstract the relevant information to the format designed for the study.

The Study format for recording individual patient details had the following information from:

- **TB treatment card**. Disease classification, history of previous TB treatment, type of patient, treatment regularity and treatment outcome.
- **ART center records**. Pre ART assessment, ART and Cotrimaxozole prophylactic treatment (CPT) initiation.
- **Patient interview**. Socio-demographic profile, previous TB treatment, HIV status of the spouse, linkage to ART center, pre ART assessment, ART and CPT initiation and history of OIs.

In addition, the time, place and cause of death of patients reported dead were obtained from reliable informants well acquainted with the patient.

Data collection was initiated in March 2009 and completed in March 2010.

### Data analysis

Data management and analysis was done using Microsoft Access 2002 and SPSS version 15.0 after checking for completeness and consistency. The categorical variables are described as numbers (%) and continuous variables as Mean [Standard Deviation (SD)] or Median [Inter Quartile Range (IQR)]. The categorical variables were assessed using Pearson chi-square test and calculating Odds Ratio (OR) with corresponding 95% Confidence Interval. Medians were compared using Mann Whitney Test.

The treatment related variables of patients with ‘**favourable**’ and ‘**unfavourable**’ treatment outcomes as well as of those **‘dead’** and **‘survived’** were compared initially through univariate analysis to identify factors associated with ‘treatment outcome’ and ‘survival status’. Logistic regression analysis was then done to estimate the independent effect of the variables that were significantly associated. Variables yielding p values<0.1 in univariate analysis were included in the logistic regression model. A backward stepwise elimination procedure based on the likelihood statistics (using probability of 0.1 for removal and 0.05 for entry) was performed to identify the best subset of variables associated with treatment outcome and survival status. Statistical tests were carried out at 5% level of significance.

## Results

**Figure 1:**
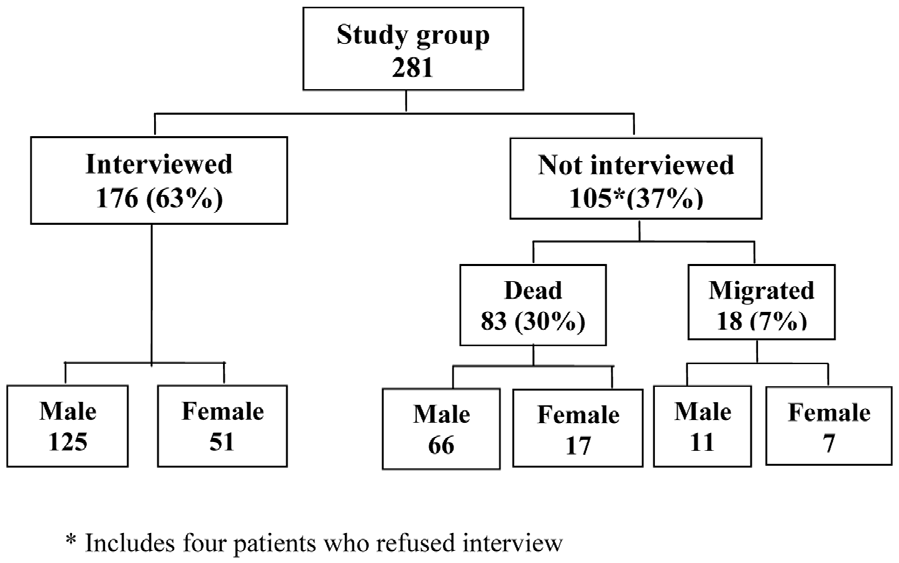
Interview coverage of the study group.

The study group of 281 HIV infected TB patients was followed up prospectively after an average period of 18.9 months from the TB treatment initiation. Of these patients, 176 (63%) could be interviewed and 83 (30%) were reported dead (figure 1).

### Personal and socio-demographic profile of patients interviewed (Table 1)

**Table 1:** Socio-demographic profile of HIV infected TB patients interviewed N = 176.

Profile	Numbers	%
**Age group**		
<15	3	2
15–44	143	81
45–64	29	16
65	1	1
**Gender**		
Male	125	71
Female	51	29
**Marital status**		
Married	143*	81
Unmarried	33	19
**Literacy**		
Literate	106	60
Illiterate	70	40
**Current employment status**		
Employed	105	60
Unemployed	51	29
Others (housewives, pensioners, dependents and students)	20	11
**Employment** **		
Labourer	55	31
Semi skilled workers	27	15
Drivers	22	13
Petty business	21	12
Agriculturist	6	3
Professionals	5	3
Others	19	9
**Residence**		
Rural	87	49
Urban	70	40
Semi-urban	19	11
**Habits**		
Alcohol	96	55
Smoking	84	48

* includes separated (10), widowed (27).

** For those employed (105).

The mean age of patients interviewed was 35 yrs (SD-9.83) – males 36 and females 33 years and 81% were in the age group of 15–44 yrs. Majority (71%) of the patients were males. Of the 143 (81%) married patients, information on HIV status of the spouse was available for 108 (76%) and of these, 61% were HIV sero-positive (not on table). Two thirds of the patients were literate and employed. Twenty nine percent of patients were currently unemployed due to illness as stated by 46 (90%) patients. The Mean duration of unemployment was 9 months ranging from 1–36 months (not on table). History of alcoholism and smoking was given by 55% and 48% of the patients respectively.

### Disease Classification and CD4 count of the study group (Table 2)

**Table 2:** Disease Classification and CD4 counts in HIV infected TB patients N = 281.

Classification		CD4 count/mm3	
Pulmonary	No. of patients	Available for	Median (IQR)
Smear positive	77	50	140 (62.75–225)
Smear negative	63*	45	116 (72–171)
**Total**	**141** **	**95**	**118 (67.5–207.5)**
**Extrapulmonary**			
Lymph node	48	38	109.5 (58.25–221.2)
Pleural effusion	39	29	153 (83–249)
Abdominal	23	22	91 (56–163.75)
TBM	17	13	109 (79–218)
Skin & Spine	2	2	111.5 (103.25–119.75)
Site not recorded	11	9	106 (69.5–290.5)
**Total**	**140**	**113**	**116 (62–219)**

IQR – Inter Quartile Range.

* Six smear negative patients had milliary tuberculosis and their median CD4 count was 120. (95–124).

** One smear status not available.

Fifty percent of 281 patients had pulmonary and the rest had Extrapulmonary TB. Of those with pulmonary TB, 55% were smear positive. Among the 63 smear negatives 6 had disseminated form of tuberculosis. The commonest form of Extra Pulmonary TB was lymph node involvement (34%) followed by pleural effusion (28%).

CD4 count at treatment initiation was available for 208 TB patients and the median CD4 count was 117/mm^3^ (IQR 64.5–218.25). In pulmonary TB patients it was 118/mm^3^ while in those with extra pulmonary TB it was 116/mm^3^.

### TB treatment outcome

The treatment success rate of smear positive patients was 62% (Table 3). The low success rate was mainly due to ‘death’ (19%) and ‘defaults’ (16%) as only 2 (3%) patients had ‘treatment failure’. The treatment success of the study group was 75%, again attributable to 15% defaults and 9% deaths. Of the 41 patients who defaulted from treatment 26 (63%) died subsequently.

**Table 3:** Treatment outcomes of HIV infected TB patients according to Disease classification N = 281.

Treatment Outcome	Favourable Outcome		Unfavourable Outcome			Total
Classification	Cured	Treatment Completed	Default	Failure	Dead	
**Pulmonary N = 141**						
Smear positive	42 (54)	6 (8)	12 (16)	2 (3)	15 (19)	77
Smear negative	-	47 (75)	12 (19)	-	04 (6)	63
Smear status not available	-	-	-	-	1	1
Sub total	42 (30)	53 (38)	24 (17)	2(1)	20 (14)	141
**Extrapulmonary N = 140**	-	117 (83)	17 (12)	-	06 (4)	140
**Total**	42 (15)	170 (60)	41 (15)	2 (1)	26 (9)	281

( ) Percentage.

The treatment related variables considered for comparison between ‘unfavourable’ and ‘favourable’ outcome were *disease classification*, *sputum smear status in pulmonary TB patients*, *type of patient*, *treatment regularity* , *initial CD4 count* and *initiation of ART & CPT* (Table 4).

**Table 4:** Univariate analysis of the factors associated with TB treatment outcomes (N = 281).

Factors	UnfavourableOutcome(N = 69)	FavourableOutcome(N = 212)	p value	OR(95% CI)
**Disease classification**				
Pulmonary	46	95		2.46
Extra Pulmonary	23	117	0.002	(1.35–4.53)
**Sputum smear status** *				
Smear positive	29	48		1.77
Smear negative	16	47	0.122	(0.80–3.94)
**Type of patient**				
Retreatment	22	22		4.04
New	47	190	0.000	(1.96–8.35)
**Regularity of treatment** **				
Irregular	40	110		1.63
Regular	21	94	0.107	(0.86–3.09)
**Baseline CD4 count /mm3** @				
≤350	24	157		0.80
>350	4	23	0.825	(0.26–3.29)
**ART initiation**				
Not initiated	56	68		9.12
Initiated	13	144	0.000	(4.48–18.88)
**CPT provision**				
Not given	46	49		6.65
Given	23	163	0.000	(3.53–12.61)

OR - Odds Ratio, CI- Confidence interval.

* Smear status is given only for those with pulmonary TB. Smear status not available for one patient.

** Excludes 16 patients for whom treatment cards were not available (8 each in unfavourable & favourable outcome).

@ CD4 count was not available for 32 and 41 patients with favourable and unfavourable treatment outcome respectively.

‘***Retreatment cases***’ had a significantly higher probability of having ‘unfavourable’ treatment outcome as compared to ‘New Cases’ [OR-4.04, CI (1.96–8.35). ***Pulmonary TB*** was also identified as a potential risk factor for ‘unfavourable outcome’ [OR 2.46, CI (1.35–4.53)]. Non initiation of ART [OR 9.12, (CI (4.48–18.88)) and of CPT [OR 6.65, (CI 3.53–12.61)] was significantly associated with ‘unfavourable’ outcome. The median CD4 count for patients with ‘unfavourable’ and ‘favourable’ treatment outcome was 118/mm^3^ (IQR 66.5–219) and 117.5/mm^3^ (IQR 64.5–218.3) respectively (not on table). Irrespective of the availability of CD4 count or eligibility for ART overall, 157 (56%) patients in the study group were initiated on ART. Sixty percent of them were initiated on ART during TB treatment (6% within 15 days and 54% after >15 days of TB treatment initiation) while, 19% received ART prior to TB treatment and 21% after completion of TB treatment.

The proportion of patients with CD4 count of ≤350/mm^3^ did not differ significantly between the two outcome groups. The CD4 count was either not done or information was not available in any of the ART records for 73 (26%) HIV infected TB patients and none of these patients received ART. However, of these 73, 36% of patients had extra pulmonary tuberculosis and were thus eligible for ART. In the remaining 208 patients with CD4 count, 195 (94%) were eligible for ART as per the NACO guidelines (181 having CD4 count ≤350/mm^3^ and 14 with >350/mm^3^ but having Extra pulmonary TB), but of the eligible, only 157 patients received ART (not on table).

In the logistic regression analysis factors independently associated with ‘unfavourable’ outcome were disease classification as ‘***Pulmonary***’ [aOR-1.96, CI (1.02–3.77)], type of patient as ‘***retreatment***’ [aOR-4.78, CI (2.12–10.76)], and ‘***not initiated on ART***’ [aOR-4.90, CI (1.85–12.96)].

### Survival status of the study group

Survival status 19 months after TB treatment initiation revealed that overall, 83 patients were reported dead; 26 while on treatment and 57 after treatment. The median survival period was the time in months between date of TB treatment initiation and the date of death which was 7.3 months (Median days 219, IQR 139–350). Treatment related factors of ‘dead’ and ‘survived’ patients considered for comparison were similar to those for ‘treatment outcome’ (Table 5). Disease classification as ‘***pulmonary TB***’ [(OR 2.21, CI (1.26–3.88)], type as ‘***retreatment cases***’ [OR 2.05, CI (1.01–4.18)] and **‘** ***treatment irregularity*** **’** [OR 2.07, CI (1.14–3.78)] were potential risk factors for death. Proportion of patients with CD4 count of ≤200/mm^3^ did not differ significantly between the two groups. The probability of ‘death’ was significantly higher among patients ‘***not initiated on ART***’ [OR 7.75, CI (4.12–14.71)] or with ‘***CPT***’ [OR 7.76, CI (4.22–14.85)]. Favourable TB treatment outcome at the end of treatment period was 36% for the ‘dead’ compared to 92% in the ‘survived’ group. (p = 0.00). The median CD4 count of ‘dead’ and ‘survived’ patients was 137.5/mm^3^ (IQR 63–199.25) and 116/mm^3^ (IQR 9.25–321.75) respectively with no significant difference between the two.

**Table 5:** Univariate analysis of the treatment related factors associated with survival status (N = 281).

Factors	Dead (N = 83)	Survived (N = 83)	P value	OR (95% CI)
**Disease classification**				
Pulmonary	53	88		2.21
Extrapulmonary	30	110	0.002	(1.26–3.88)
**Smear status** *				
Smear positive	32	45		1.53
Smear negative	20	43	0.23	(0.72–3.26)
**Type of patient**				
Retreatment	19	25		2.05
New	64	173	0.03	(1.01–4.18)
**Regularity of treatment** **				
Irregular	53**	97**		2.07
Regular	24	91	0.01	(1.14–3.78)
**Baseline CD4 count /mm^3^** #				
≤200	23	129		0.59
>200	13	43	0.17	(0.26–1.35)
**ART initiation**				
Not initiated	64	60		7.75
Initiated	19	138	0.000	(4.12–14.71)
**CPT provision**				
CPT not given	55	40		7.76
CPT given	28	158	0.000	(4.22–14.35)
**TB treatment outcome**				
Unfavourable	53	16		20.1
Favourable	30	182	0.000	(9.70–42.10)

OR - Odds Ratio, CI- Confidence interval.

* Smear status is given only for those with pulmonary TB. Smear status not available for one patient,

** Excludes 16 patients for whom treatment cards were not available (6 in dead and 10 in survived).

# For 47 and 26 CD4 counts were not available in dead and survived patients respectively.

In the logistic regression analysis the treatment related factors independently associated with death were ‘***non initiation ART***’ [aOR-2.80, CI (1.15–6.81)] and ***of ‘CPT’*** [aOR-3.46, CI (1.47–8.14)] (Table 6).

**Table 6:** Association of treatment related risk factors with TB treatment outcome and with survival status.

Factors	Treatment outcomeunfavourable v/s favourable			Survival statusDead v/s Survived		
	aOR	95% CI	P value	aOR	95% CI	P value
**Type of patient**Retreatment/New	4.78	2.12–10.76	0.000	1.94	0.89–4.21	0.093
**Disease classification**Pulmonary/Extra pulmonary	1.96	1.02–3.77	0.044	1.82	1.00–3.33	0.050
**ART initiation**Not on ART / on ART	4.90	1.85–12.96	0.001	2.80	1.15–6.81	0.023
**CPT provision**Not initiated/initiated	2.19	0.90–5.32	0.083	3.46	1.47–8.14	0.004
**Prediction**	**80.8%**			**76.5%**		

aOR- Adjusted Odds Ratio, CI – Confidence Interval.

## Discussion

We evaluated the treatment outcome and survival status 19 months after TB treatment initiation of a cohort of HIV infected TB patients from a high HIV prevalent district of South India. Only 63% of the patients could be interviewed. The main reason for attrition in the coverage was a high proportion of deaths.

Patient profile revealed that majority (81%) were in the socio-economically productive age group of 15–44 yrs and were married. A high proportion (61%) of spouses being infected with HIV reemphasises the importance of disclosing HIV status of the patient to the spouse at the post-test counselling and the necessity for routinely monitoring HIV status of the spouse. This also gives a unique opportunity for positive prevention in a high proportion (about 40%) of couples discordant for HIV by reinforcing their awareness on precautions to be taken to prevent transmission of infection. The current unemployment due to illness in one third of the patients in the economically productive age group is likely to affect the families already in the lower socio-economic strata. More than half of the patients were habituated to alcohol and smoking indicates their vulnerability to undesirable habits.

Pulmonary and extra pulmonary TB occurred with same frequency in the cohort in contrast to other reported studies, wherein higher proportion (50–72%) of HIV infected TB patients had extra pulmonary TB [22]–[25]. The probable reason for this difference could be missing/underreporting of extrapulmonary TB cases by the peripheral health centres due to limited diagnostic facilities. Lymph node was the commonest site involved as observed in previous studies too [24],[26].

Majority (73%) of the 208 patients in the study group had a CD4 count of <200/mm^3^ indicating progressive immunodeficiency. Manifestation of extra pulmonary TB in HIV sero-positives denotes a greater degree of immunosuppression. However, as also reported in earlier studies, the median CD4 count in patients with various forms of extra pulmonary TB though lower than in pulmonary TB did not differ significantly, suggesting that clinical presentation of tuberculosis did not correlate with CD4 count [23], . In contrast, Brenda et.al have reported a progressive increase in the frequency of extra pulmonary TB as CD4 cell count decreases suggesting that CD4 cells play a major role in limiting the severity of TB [26].

The treatment success of smear positive HIV infected TB patients treated with intermittent SCC regimens in our study was lower than that in TB patients irrespective of HIV status reported by the programme for the district during the corresponding period (61% v/s 80%) [29]. The lower treatment success was attributable to 19% deaths and 15% defaults. Favourable response of 72% has been reported from south India among culture positive pulmonary TB cases with HIV treated with intermittent SCC [18]. Treatment completion rate of 65–73% with SCC among HIV infected TB patients has also been reported from West Africa, which was mainly due to high mortality [13]. The high default rate observed in our cohort could perhaps be due to patients stop attending for treatment due to ill health or other reasons like work commitment , alcoholism etc. and requires further studies.

In the logistic regression analysis ***Retreatment cases*** were found to be associated with unfavourable treatment outcome. Even though drug susceptibility test was not done in our study, prior sub optimal therapy is known to be a major contributor to the development of Multi Drug Resistant (MDR) TB [23]. Normally MDR confers a high mortality rate for both HIV seropositive and seronegative patients [24], [30]. In addition, HIV infected patients having MDR TB were twice as likely to die than those who did not have MDR TB [31].

***Non initiation of ART*** has also emerged as a risk factor for ‘unfavourable’ treatment outcome. Appropriate time of ART initiation, while on TB treatment has been an issue of dispute. Of the 157 (56%0 patients in the study group initiated on ART ; 19% were already on ART and only 6% were initiated within 15 days of TB treatment as per NACO guidelines. Studies have demonstrated that simultaneous use of ART with anti TB treatment significantly reduces the risk of death both short and long term [32], [33]. Hence, the pros and cons of early initiation of ART especially for those with advanced immunosuppression should be assessed carefully.

Although, extra pulmonary tuberculosis is generally associated with higher degree of immunosuppression, **pulmonary tuberculosis** has emerged as a risk factor for ‘unfavourable outcome’ in our study as well as in Kenya in contrast to other report where it was not so [34].

A high mortality (30%) observed in our study has also been reported in several studies among HIV infected TB patients [13], [18], [24], [27], [35]. Nearly four times higher death rates were observed in HIV positive compared to HIV negative TB patients during the first six months after treatment initiation in a prospective cohort study in Kenya [15]. The median survival time was 7.3 months in our study which was similar to that reported from San Francisco (7.4 months) [24] and Peru (9 months) [34]. The higher mortality in HIV associated tuberculosis could be mostly due to other OIs, which occur in the presence of profound immunosuppression as also seen in our study group wherein the median CD4 count was 117/mm^3^. Tuberculosis is known to accelerate HIV disease progression because of production of cytokines like TNF-α, which increases viral replication.

*Patients with pulmonary TB, retreatment cases, with treatment irregularity, not receiving ART & CPT and those with unfavourable TB treatment outcome* were the factors associated with mortality in the univariate analysis. However in the logistic regression analysis ‘***non initiation of ART***’ and of ***‘CPT’*** emerged as strong risk factors for mortality. Surprisingly, lower CD4 count of <200/mm^3^ indicating advanced immunosuppression was not associated with mortality in our study is reported by others too, possibly because TB itself may enhance the progression of HIV infection [15], [17], [34], [36], [37]. This is in contradiction with the higher deaths reported among patients with baseline CD4 counts of <200/mm^3^ [32], [35]. The probable explanation for this difference could be the non availability of CD4 count for a substantial proportion (56%) of dead patients.

Proportion of patients with irregular drug intake and those having unfavourable TB treatment outcome particularly, defaults were higher in the ‘dead’ group. Receipt of inadequate anti TB treatment has shown to increase the risk of death and is consistent with other reports [23], [38].

There is adequate data to show that patients with TB and HIV who did not receive ART had a short survival time [31], [32], [39]. The reduction in the risk of death with addition of ART has been reported from Peru and San Francisco [34], [40]. Despite the apparent benefit associated with ART only 56% of the HIV infected TB patients in our study and 44% in San Francisco received ART. Although, deferring ART simplifies the management of two diseases, the results provide compelling evidence to warrant initiation of ART along with TB treatment. Unfortunately, most HIV infected TB patients in many developing countries still cannot access ART primarily due to economic barriers and limited coverage. If ART is deferred another OI may superimpose and accelerate HIV disease progression [41]. The importance of chemoprophylactic treatment to prevent other OIs may also play a significant role in avoiding deaths particularly when the access to ART is limited as in developing countries [39].

Even in accordance to NACO ART guidelines, 80% of the eligible TB patients with CD4 count available received ART in our study [42]. The important contributing factor for this gap could be the provision of ART from a single centre situated in a tertiary health care setting in the district during the study period. However, expansion of ART facilities is now under progress particularly in high HIV prevalent areas. TB treatment outcomes did not differ significantly among those with CD4 count of >350/mm^3^ compared to those with <350/mm^3^ and over 94% of patients with CD4 count available were anyway eligible for ART as per current National guidelines. Moreover, ‘*non initiation of ART*’ was significantly associated with mortality. This makes a strong case for changing the current National guidelines to align with WHO recommendation that all HIV infected TB patients should be initiated on ART irrespective of CD4 counts.

Being a retrospective study conducted under programme conditions and based on the information from RNTCP and NACP programme records and patient interviews, was subjected to certain limitations. Firstly, some of the important potential factors including clinical manifestations, radiological presentation, drug susceptibility status and occurrence of OIs which are likely to influence the TB treatment outcome or survival status could not be studied. Secondly, despite repeated efforts by the study team, the CD4 cell count was not available for a substantial proportion (26%) of patients from the ART records, particularly, for those defaulted and dead. This could have added to the knowledge on the association between immunosuppression and unfavourable outcome. Finally, the exact cause of death could not be ascertained in many patients as majority of the deaths occurred outside the hospital and relevant records for the same were not available with the informants.

### Conclusion

Despite the availability of effective treatment for TB and HIV, the high mortality observed in the cohort is a matter of concern and needs early intervention. The study highlights the importance of preventing premature cessation of anti-TB treatment leading to default wherein the proportion of death was high. Non initiation of ART has emerged as a high risk factor for unfavourable treatment outcome as well as for overall mortality. Also, CPT to prevent other OIs may play a role in avoiding deaths particularly, when ART is not initiated. These findings underscore the importance of access and availability of ART services as a priority, especially in high HIV prevalent areas. This also makes a strong case for reconsidering current programme guidelines on ART initiation to make it in line with WHO recommendation of initiating ART to all TB patients irrespective of CD4 count.

Treatment with standard anti TB regimens using DOTS could be effective in improving the survival and quality of life of patients with HIV and TB, provided there is concomitant ART to prevent continued immunological deterioration and progression of HIV. More Studies are needed to assess the efficacy of SSC intermittent regimen and their long term outcome, particularly, mortality and relapses.

## References

[1] Narain JP, Raviglione MC, Kochi A (1992). HIV associated tuberculosis in developing countries: Epidemiology and strategies for prevention.. Tuberc Lung Dis.

[2] Singh A, Bairy I, Shivananda PG (2003). Spectrum of opportunistic infections in AIDS cases.. Indian J Med Sci.

[3] Girardi E, Raviglione MC, Antonucci G (2000). Impact of the HIV epidemic on the spread of other diseases: the case of tuberculosis.. AIDS.

[4] Corbett EK, Watt CJ, Walker N, Maher D, Williams BG (2003). The growing burden of tuberculosis: global trends and interactions with the HIV epidemic burden of tuberculosis.. Arch Intern Med.

[5] Harries AD (1990). TB and HIV in developing countries.. Lancet.

[6] Raviglione MC, Narain J, Kochi A (1992). HIV and TB in developing countries. Clinical features, diagnosis and treatment.. Bull WHO.

[7] Paranjape RS, Tripathy SP, Menon PA, Joshi DR, Patil U (1997). Increasing trend of HIV seroprevalence among pulmonary tuberculosis patients in Pune, India.. Ind J Med Res.

[8] World Health Organization, Global tuberculosis Control: WHO report (2010).

[9] National AIDS Control Organization, Department of AIDS Control, Annual report 2009–10: Ministry of Health and Family Welfare, Government of India

[10] Vijay S, Swaminathan S, Vaidyanathan P, Thomas A, Chauhan LS (2009). Feasibility of provider-initiated HIV testing and counselling of tuberculosis patients under the TB control programme in two districts of South India.. PLoS ONE.

[11] Rana FS, Hawken MP, Mwachari C (2000). Autopsy study of HIV-1-positive and HIV-1-negative adult medical patients in Nairobi, Kenya.. J Acquir Immune Defic Sundr.

[12] Corbett EL, Churchyard GJ, Charalambos S (2002). Morbidity and mortality in South African gold miners: impact of untreated disease due to human immunodeficiency virus.. Clin infect Dis.

[13] Kassim S, Sassan-Morokro M, Ackah A, Abouya LY, Digbeu H (1995). Two year follow up of persons with HIV-1 and HIV-2 associated pulmonary tuberculosis treated with short-course chemotherapy in West Africa.. AIDS.

[14] Colebunders RL, Ryder RW, Nzilambi N (1989). HIV infection in patients with tuberculosis in Kinshasa, Zaire.. Am Rev Respir Dis.

[15] Nunn P, Brindle R, Carpenter L, Odhiambo J, Wasunna K (1992). Cohort study of human immunodeficiency virus infection in patients with tuberculosis in Nairobi, Kenya.. Am Rev Respir Dis.

[16] Lucas SB, Hounnou A, Peacock C (1993). The mortality and pathology of HIV infection in a West African city.. AIDS.

[17] Small P, Schecter G, Goodman P, Sande M, Chaisson R (1991). Treatment of tuberculosis in patients with advanced human immunodeficiency virus infection.. N Engl J Med.

[18] Swaminathan S, Deivanayagam CN, Rajasekaran S, Venkatesan P, Padmapriyadarsini C (2008). Long term follow up of HIV-infected patients with tuberculosis treated with 6-month intermittent short course chemotherapy.. The National Medical Journal of India.

[19] HIV Sentinel Surveillance and HIV Estimation, 2006 (2008).

[20] National AIDS Control Organization (2008). Operational Guidelines for Integrated Counselling and Testing Centres.

[21] Technical and Operational Guidelines for Tuberculosis Control (2005). http://www.tbcindia.org/pdfs/Technical&OperationalguidelinesforTBControl.pdfs.

[22] Purohit SD, Gupta RD, Bhatura VK (1996). Pulmonary tuberculosis and human immunodeficiency virus infection in Ajmer.. Lung India.

[23] Alpert PL, Munsiff SS, Gourevitch MN, Greenberg B, Klein RS (1997). A prospective study of tuberculosis and HIV, clinical manifestations and factors associated with survival.. Clin Infect dis.

[24] Chaisson RE, Schecter GF, Theuer CP, Rutherford GW, Echenberg DF (1987). Tubercuosis in patients with the acquired immunodeficiency syndrome-clinical features, response to therapy and survival.. Am Rev Respir Dis.

[25] Pitchenik AE, Cole C, Russell BW, Fischi MA, Spira TJ, Snider DE (1984). Tuberculosis, atypical mycobacterosis, and the acquired immunodeficiency syndrome among Haitain and non-Haitian patients in South Florida.. Ann Intern Med.

[26] Jones BE, Young SMM, Antoniskis D, Davidson PT, Kramer F, Barnes PF (1993). Relationship of the manifestations of tuberculosis to CD4 cell counts in patients with human immunodeficiency virus infection.. Am Rev Respir Dis.

[27] Ackah AN, Coulibaly D, Digbeu H, Diallo K, Vetter KM, Coulibaly IM (1995). Response to treatment, mortality, and CD4 lymphocyte counts in HIV-infected persons with tuberculosis in Abidjan, Côte d'Ivoire.. Lancet.

[28] Llibre JM, Tor J, Manterola JM, Carbonell C, Roset J (1992). Risk stratification for dissemination of tuberculosis in HIV-infected patients.. Quart J Med.

[29] Central TB Division (2010). RNTCP Performance Report, India: Second Quarter 2010.

[30] Quy HT, Cobelens FG, Lan NT (2006). Treatment outcome by drug resistance and HIV status among Tuberculosis patients in Ho Chi Minh City, Vietnam.. Int J Tuberc Lung Dis.

[31] Manosuthi W, Chottanapand S, Thongyen S, Chaovavanich A, Sungkanuparph S (2006). Survival rate and risk factors of mortality among HIV/tuberculosis-coinfected patients with and without antiretroviral therapy.. Journal of Acquired Immune deficiency Syndromes September.

[32] Dheda K, Lampe FC, Johnson MA, Lipman MC (2004). Outcomes of HIV– associated Tuberculosis in the era of highly active Antiretroviral therapy.. J Infect Dis.

[33] Dean GL, Edwards SG, Ives NJ, Mathews G, Fox EF (2002). Treatment of tuberculosis in HIV-infected persons in the era of HAART.. AIDS.

[34] Collins JA, Alarcon JO, Moore DAJ, Hernandez AV, Salazar R (2010). Effect of antiretroviral therapy on survival of HIV infected tuberculosis patients in Peru.. Rev Panam Infectol.

[35] Whalen C, Nsubuga P, Okwera A, Johnson JL, Hom DL (2000). Impact of pulmonary tuberculosis on survival of HIV infected adults, a prospective epidemiological study in Uganda AIDS.

[36] Theuer CP, Hopewell PC, Elias D, Schecter GF, Rutherford G (1990). Human immunodeficiency virus infection in tuberculosis patients.. J Infect Dis.

[37] Chaisson R, Hopewell P (1990). Survival after active tuberculosis in patients with HIV infection.. Am Rev Respir Dis.

[38] Leroy V, Salmi RL, Dupon M, Sentilhes A, Texier- Maugein J, Dequae L (1997). Progression of human immunodeficiency virus infection in patients with tuberculosis disease. A cohort study in Burdeaux, France. 1998–1994.. Am J Epidemiol.

[39] Murray J (1991). Tuberculosis and human immunodeficiency virus infection during the 1990s.. Bull int Union Tuberc Lung Dis;.

[40] Nahid P, Gonzlez LC, Rudoy I, Bouke C, de Jong (2007). Treatment outcomes of patients with HIV and tuberculosis.. American journal of respiratory and critical care medicine.

[41] Badri M, Ehrlich R, Wood R (2001). Association between tuberculosis and HIV disease progression in a high tuberculosis prevalence area.. Int J Tuberc Lung dis.

[42] National AIDS Control Organization (June 2008). Training Manual on Intensified TB/HIV Package for Medical Officers.

